# Treating SOD1-ALS with tofersen results in nonprogressive chronic ALS—a case series from Iceland

**DOI:** 10.1007/s00415-025-13579-y

**Published:** 2026-02-11

**Authors:** Bjorn Logi Thorarinsson, Olafur Arni Sveinsson, Agust Hilmarsson, Thora Bjorg Sigurthorsdottir, Peter Munch Andersen

**Affiliations:** 1 Landspitali University Hospital Reykjavik, Skaftahlid 24, 105 Reykjavik, Iceland; 2 Faculty of Medicine, University of Iceland, Reykjavik, Iceland; 3 Department of Clinical Sciences, Neurosciences, Umeå University, 901 87 Umeå, Sweden

**Keywords:** ALS, Superoxide dismutase 1, Antisense oligonucleotide, Neurofilament light chain, Tofersen

## Abstract

Amyotrophic lateral sclerosis (ALS) is a progressive neurodegenerative disorder. We describe four patients with hereditary ALS caused by the p.Gly94Ser *SOD1* mutation who were treated monthly with the intrathecal antisense oligonucleotide tofersen in a clinical setting at Landspitali University Hospital of Iceland. After initiating treatment 15–26 months ago, no significant clinical deterioration was observed, and three patients showed signs of clinical improvement, with some recovery of motor function. All four patients currently present with chronic nonprogressive ALS, a phenotype not previously observed or documented. Concomitantly, the concentration of neurofilament light chain (Nf-L) in the cerebrospinal fluid decreased to the normal range. This clinical benefit and decrease in Nf-L levels were detected regardless of the patient’s initial ALSFRS-R score. No serious adverse events were observed. Notably, we observed a clinically meaningful effect in two patients who had been ill for several years before treatment was instituted, raising questions about who should receive treatment and the biology of paresis and motor neuron cell loss in patients with ALS. Although only a minority of ALS patients carry a *SOD1* mutation, the advent of this new precision medicine has profound implications for ALS management.

**Supplementary Information:**

The online version contains supplementary material available at 10.1007/s00415-025-13579-y.

## Introduction

Amyotrophic lateral sclerosis (ALS) is a fatal adult-onset neurodegenerative disorder that primarily affects motor neurons innervating striated muscle, resulting in progressive weakness and, eventually, generalized paralysis. Symptoms typically begin focally in one myotome and progress to adjacent myotomes, eventually involving most striated muscles, resulting in generalized paresis and weakness of the respiratory muscles leading to death [1].

ALS is now recognized as a syndrome with heterogeneous aetiology and genetic variants, clinical presentation, prognosis and neuropathology. Mutations in some 40 genes have been associated with ALS [2]. Missense and nonsense variants in the small gene encoding the constitutively and ubiquitously expressed superoxide dismutase type 1 (SOD1) are globally the most common cause of hereditary ALS (HALS). This accounts for 1 to 23% of familial cases and 1–2% of patients with a sporadic ALS diagnosis [3]. In Iceland, *SOD1* mutations represent the most frequent cause of HALS (manuscript in preparation). More than 234 variants in *SOD1* have been identified in ALS patients worldwide; most are inherited as a dominant trait with complete or reduced penetrance, but recessive inheritance and cases with de novo mutation have also been reported. The most common variant in Iceland is c.280G > A, p.Gly94Ser. Both missense and nonsense mutations in *SOD1* have been demonstrated to be pathogenic for ALS. Taken together, these findings suggest that the disease mechanism involves misfolding of the immature SOD1 polypeptide to form prion-like protein species capable of propagating from the origin of initiation throughout the motor axis [4]. Postmortem studies have revealed cytoplasmic aggregates containing misfolded SOD1 in motor neurons. Inoculation of such aggregates purified from the spinal cord of a patient with p.Lys128GlyfsTer6 (also known as G127X *SOD1*) into the ventral horn of 100-day-old transgenic mice resulted in premature disease in a *dose-dependent* manner 60–80 days later, with all the features of human ALS (focal asymmetrical onset, spread to contiguous myotomes and eventual generalized atrophy and paresis) with the concomitant appearance of cytoplasmic aggregates of misfolded SOD1 [4]. This finding and other studies support the hypothesis that the concentration of the SOD1 monomer is critical for the development of ALS.

Effective treatments for ALS are lacking. The emerging understanding of the underlying pathophysiology of mutant SOD1-mediated ALS has led to the development of tofersen, a 20-base antisense oligonucleotide (ASO) that binds to SOD1 mRNA and triggers RNase H-mediated degradation of the mRNA, and hence a reduction in SOD1 synthesis. Tofersen is administered intrathecally every four weeks and has been assessed in double-blind, randomized controlled trials and in open-label, Phase IV studies. Initially, a Phase I–II trial demonstrated that it was well tolerated and significantly reduced the levels of SOD1 protein in cerebrospinal fluid (CSF) and neurofilament L (Nf-L) in blood and CSF [5]. A subsequent Phase III study, VALOR, did not significantly improve the primary clinical endpoint (the ALSFRS-R functional rating score) after 28 weeks. However, reductions in CSF SOD1 protein and Nf-L concentrations were observed in the treatment arm, indicating target engagement and biological effects of the intervention [6]. All participants were later offered enrolment in a longer open-label extension study, where sustained reductions in SOD1 and Nf-L levels were observed after 52 weeks in the group receiving early treatment, and a significant reduction was detected in those who transitioned from placebo. Clinically meaningful differences in muscle strength and pulmonary function were observed after 52 weeks between the early and delayed treatment groups [6]. In response to these results, the drug was approved by the U.S. Food and Drug Administration in April 2023 [7] and by the European Medicines Agency in May 2024 [8].

At Landspitali University Hospital Reykjavik, tofersen treatment began in March 2023 through the early access program offered by Biogen [9, 10]. All four symptomatic patients in Iceland presenting with *SOD1* c.280G > A, p.Gly94Ser-mediated ALS were initiated on tofersen therapy. With patients’ consent, we describe the outcomes of all Icelandic patients treated with tofersen between 2023 and 2025. There were no treatment failures. At the end of the early access program in July 2025, all four continued treatment.

All assessments were conducted by the same neurologists and physiotherapist under standardized conditions. The first assessment (M1) was performed immediately before or after the initial drug administration (Initial Dose, ID) in all participants. The study was approved by the National Bioethics Committee in Iceland (VSN-23–116).

## Case 1

**Fig. 1:**
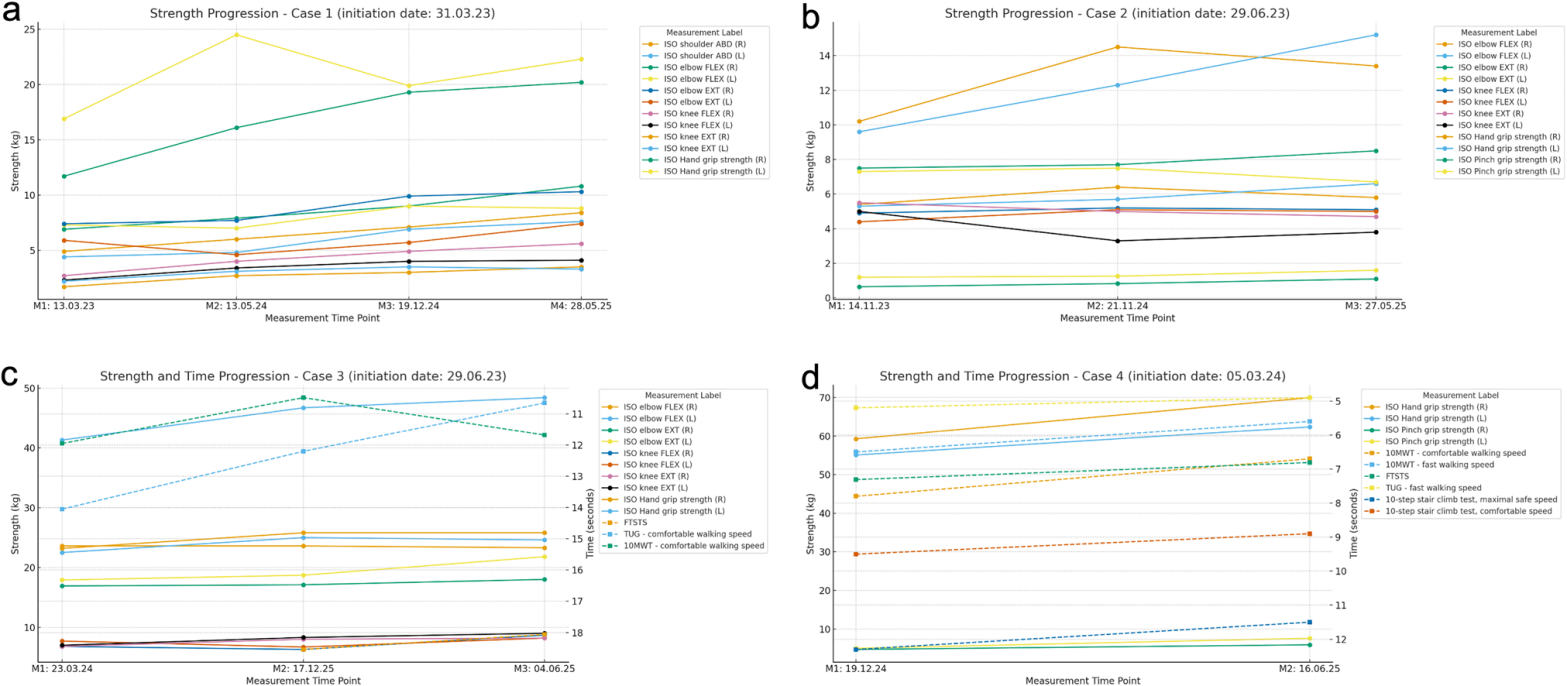
Longitudinal assessment of motor strength and functional mobility by a physiotherapist. Measurements in the four patients: 1a) Case 1: First physiotherapist assessment 13.03.2025, tofersen start 31.3.2023, reassessments: 19.12.2024 and 28.05.2025; 1b) Case 2: Tofersen start 29.6.2023, physiotherapist assessments: 14.11.2023, 21.11.2024 and 27.05.2025; 1c) Case 3: Tofersen start 29.6.2023, physiotherapist assessments: 23.03.2024, 17.12.2024 and 03.06.2025; Case 4: Tofersen start 5.3.2024, physiotherapist assessments: 19.12.2024 and 16.06.2025

**Fig. 2:**
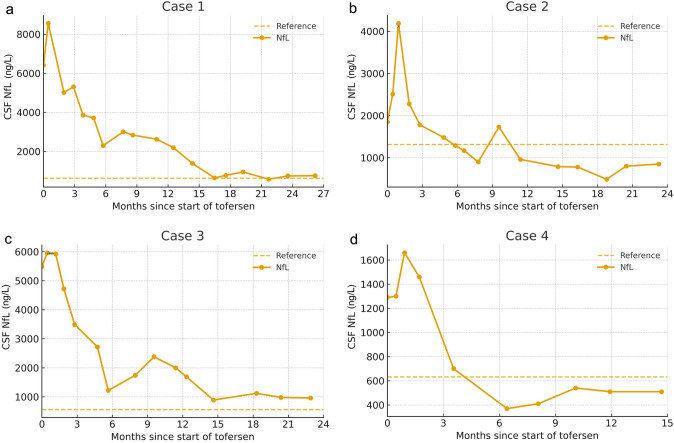
Neurofilament light chain levels in cerebrospinal fluid before and during treatment with tofersen. Figure 2a–d Nf-L concentration in the CSF during treatment with tofersen. The origo on the X-axis correlates with the first tofersen injection. In all four patients, the decline approached a steady state after approximately six months of treatment, and no further reduction was observed. Nf-L levels decreased by 89.9% in Case 1 (from 7485 to 755 pg/ml), 71.1% in Case 2 (from 2850 to 825 pg/ ml), 83.7% in Case 3 (from 5960 to 970 pg/ml), and 64.0% in Case 4 (from 1417 to 510 pg/ml). Baseline values at the start of tofersen treatment were calculated as the mean of measurements taken during the first month of treatment, while response values were defined as the mean of the last two measurements during the treatment period. The reference values for cerebrospinal Nf-L are ageadjusted and are higher in case 2 (< 1314 ng/L) than in Cases 1 and 4 (< 632 ng/L) and Case 3 (< 398 ng/L)

A 47-year-old woman had a four-year history of progressive weakness in the lower limbs starting on the left, progressing to paresis and atrophy in the arms twelve months before starting tofersen. Mild dysphonia and dyspnoea appeared two months before the start of treatment. She could only walk a few steps with a high walker; otherwise, she required a wheelchair. Neurological examination revealed lower motor neuron (LMN) involvement, more prominent in the legs than in the arms. A Babinski sign was observed bilaterally. The patient was treated with riluzole for 6 weeks after being diagnosed with ALS, but the treatment was stopped because of tolerability issues. Tofersen was started in March 2023 when the patient had an ALSFRS-R score of 34 and 4/5 on the Kings Staging System (KSS) [11]. After 21 months of treatment, she no longer experienced dysphonia or shortness of breath. Improvements in activities of daily living (ADLs) were also observed. She could get out of bed more easily and walk farther with the walker, and she no longer needed a turning sheet at night to turn herself in bed. She could dress herself again unassisted and had increased strength in the right hand, allowing her to comb her hair, cook, and eat independently. Testing by a physiotherapist showed significant improvements in strength in nearly all measurements. Further improvement was observed after 26 months of treatment, with an even better ability to ambulate with a walker. She thus became fully independent in her ability to perform ADLs after 26 months of treatment, and both the ALSFRS-R and the KSS improved to 38 and 2/5, respectively. The improvement she noted in muscle strength in all four limbs was supported by an assessment by a physiotherapist (Fig. 1a) along with a consistent longitudinal reduction in Nf-L levels in the CSF (Fig. 2a).

## Case 2

A 73-year-old man had an eight-year history of steady progressive weakness beginning on the left and then involving both legs and later progressive weakness in both arms. Bulbar involvement with dysarthria and dysphagia had appeared five years prior to starting tofersen. Neurological examinations revealed predominantly progressive LMN involvement, which was more prominent in the legs but also involved the arms and bulbar region. Three years after onset in the legs, night-time noninvasive ventilation was instituted, and a gastrointestinal feeding tube was inserted. He ambulated in a wheelchair and received home nursing at the start of tofersen treatment in June 2023. The ALSFRS-R was 28. After 10 months of treatment, the need for night-time ventilatory support decreased, and inspiratory positive airway pressure (IPAP) was lowered from 16 to 15 cm H_2_O. After 17 months of treatment, bulbar improvement was noted with an objectively stronger voice and better respiratory function, and he had regained the ability to hum to music, which he had lost previously. After 23 months of treatment, his dysarthria had noticeably decreased, and he can now easily speak for 20 min without dyspnoea. Furthermore, the BiPAP pressure levels have been reduced from 16/11 cm H_2_O to 13/9 cm H_2_O over the 23 months of treatment. He also feels improvement in distal arm strength, e.g., able to open milk containers. Handwriting is easier, and he can adjust his wristwatch without help. Using his arms, he can now independently move to and from his wheelchair. The improvement he noted in the arms was supported by an assessment of muscle strength by a physiotherapist (Fig. 1b). His ALSFRS-R score improved to 32, but his KSS score was stable at 4/5. The CSF Nf-L levels show a consistent reduction over time (Fig. 2b).

## Case 3

A 36-year-old man had a seven-year history of progressive weakness, starting distally in both legs and progressing to both arms nearly 3 years before starting tofersen. Walking ability deteriorated from unassisted to walking with two canes, accompanied by reduced grip strength. He had no symptoms or signs of bulbar or respiratory involvement. He was independent in ADLs and drove a car.

Tofersen was initiated in June 2023 when the ALSFRS-R was 43. After 23 months of treatment, his condition has remained stable with no worsening of existing symptoms, and no new paresis has appeared. The apparent stabilization in leg and arm muscle function was supported by assessments after 18 and 23 months of treatment. While some already affected muscles showed slight improvements and others showed slight worsening, these changes did not result in any significant overall improvement or worsening (Fig. 1c). Both his ALSFRS-R and KSS scores were stable at 43 and 2/5, respectively. A consistent reduction in Nf-L levels in the CSF was observed (Fig. 2c).

## Case 4

A 42-year-old man had a 27-month history of progressive distal lower limb weakness. The patient had difficulty standing on the tiptoes, and widespread fasciculations were present. Three months prior to starting tofersen, he had completed a 48-week clinical trial in which an antibody against misfolded SOD1 protein was tested (NCT05039099). During and after the trial, he experienced slow, continued progression of symptoms (details classified).

Tofersen was initiated in March 2024, with the ALSFRS-R score at 44. After nine months of treatment, he reported improved walking endurance, less fatigue and increased strength in the legs, with an improved ability to walk longer distances. Eventually, he was able to return to a very demanding job full-time after having been on sick leave for more than a year prior to the tofersen treatment. Fifteen months after initiating treatment, his walking and endurance further improved. He can walk a defined 1.8 km long route in 20 min instead of 26 min before the treatment and had recently gone on a mountain hike with a 575 m elevation that he had not been able to do since becoming ill. Physiotherapy assessments supported this, as his performance on the 6-min walking test was 11.7% better than tests performed after 9 and 15 months of treatment (Fig. 1d). A consistent reduction in Nf-L levels in the CSF was observed (Fig. 2d). His ALSFRS-R score improved to 46, but his KSS score remained stable at 1/5.

## Discussion

Here, we report the first four patients in Iceland with hereditary ALS due to the *SOD1* c.280G > A, p.Gly94Ser variant who received treatment with tofersen. In three of the four patients, subjective improvement correlated concurrently with objective improvement in muscle strength and noninvasive pulmonary assistance. Nf-L levels decreased in all patients beginning approximately 4 months after the initiation of treatment; in some cases, the decrease was drastic, reaching the normal range observed in healthy individuals. No serious side effects were observed after 103 injections during the combined 87-month treatment in four patients as of June 2025. All patients are now clinically stable or have shown improvement in the previously affected limbs, with no signs or symptoms of further disease progression or development of new paresis after the initiation of treatment. This finding contrasts with the steady deterioration observed in these patients before the initiation of treatment and the observed disease course in their currently deceased, affected family members. Disease arrest in ALS, as observed here, has never been reported in the Icelandic ALS population. This new clinical condition may be denoted as chronic stable ALS (CS-ALS) to distinguish it from the untreated condition, which can be termed progressive ALS (P-ALS).

While the pivotal VALOR trial did not demonstrate a statistically significant improvement in the primary outcome measure ALSFRS-R, the placebo-controlled component was brief, and the secondary variables showed a general trend towards a positive effect. This trend was strengthened for all the variables in the follow-up open-label extension (OLE) study, which extended for several months or years, depending on when the patient was enrolled in VALOR. Notably, patients who received early treatment had more favourable outcomes in terms of muscle strength and respiratory function compared to those who initially received placebo. Our open-label experience aligns with these results, i.e., the patients who receive early treatment have the most favourable outcomes. In our unselected cases, no further disease progression of involved myotomes or involvement of new myotomes was noted after treatment started; stabilization or improvement was seen irrespective of the patient’s age or stage of disease. Perhaps not surprisingly, a clinical benefit was first seen in the myotome segments that had most recently become affected before treatment initiation. These observations suggest that early intervention with tofersen is most beneficial, but later treatment can also be beneficial for preserving meaningful motor function, irrespective of disease stage and altering the natural disease course.

The primary biomarker for monitoring treatment response in patients with ALS is the concentration of Nf-L in the CSF [3]. All four cases had typical elevations in CSF-Nf-L levels, but to variable levels. Their Nf-L levels began to decrease after 2–4 months of treatment and remained at or below the upper reference limit. These results support the biological mode of action of tofersen and its potential to reduce motor neuron damage. The correlation between Nf-L levels and clinical course has not yet been fully established, but the present observations support that normalization of Nf-L represents a promising surrogate marker of successful treatment response.

The patients described here all carry the same pathogenic *SOD1* variant p.Gly94Ser. The disease course and clinical features in our cohort prior to treatment with tofersen are in accordance with previous descriptions of ALS patients with mutations in codon 93; i.e., these patients often present with lower limb onset and predominantly LMN signs and rapid progression [12]. All our patients showed a favourable response at the same standard dosage of 100 mg/month, but with different response times (Fig. 1a–d). Three patients showed clinically meaningful improvement in functions on standard treatment dose, and tolerability in all four patients was good. This raises the question of whether tofersen dosing should be personalized; for example, should some patients receive a higher dose to achieve clinical benefit instead of the limited goal of only halting disease progression? A recent study revealed large variance in SOD1 content in the CSF even in ALS patients with the same *SOD1* mutation, suggesting that dosing with SOD1-reducing drugs should be personalized [13].

A limitation of our study is the number of patients and its open-label design. Nonetheless, this is outweighed by several factors. The patient group is well defined, with all patients belonging to the same founding population and having the same *SOD1* mutation, and all were managed and assessed by the same clinical care team; this includes patients with *SOD1* mutation assessed before tofersen became available. Additionally, the long follow-up period of 15–26 months with consistent clinical and Nf-L efficacy strengthens the conclusions. Finally, Nf-L analysis was performed in the same accredited laboratory with over 20 years of experience [14]. When tofersen first became available as an EAP in Iceland in 2023, all the diagnosed patients were offered treatment without any restrictions. Recently, a fifth patient with ALS due to p.Gly94Ser *SOD1* was diagnosed and is currently receiving treatment. In conclusion, tofersen represents a novel disease-modifying therapy for the subset of patients with ALS caused by *SOD1* mutations.

## Supplementary Information

Below is the link to the electronic supplementary material.

Supplementary file1 (DOCX 15 KB)

## Data Availability

The anonymised patient-level data supporting the findings of this study, including cerebrospinal fluid neurofilament light chain (CSFNfL) measurements and physiotherapist-assessed muscle strength data, are not publicly available due to the sensitive nature of the data and patient confidentiality. The data may be made available upon reasonable request to the corresponding author, subject to informed consent from the patients and approval by the relevant ethics committee.
