# Supplementary material for: Treating SOD1-ALS with tofersen results in nonprogressive chronic ALS—a case series from Iceland
Source: J Neurol. 2026 Feb 11;273(2):140. doi: 10.1007/s00415-025-13579-y (PMC12894152; doi:10.1007/s00415-025-13579-y)
Supplement: Supplementary file 1 — Supplementary file1 (DOCX 15 KB) [file 415_2025_13579_MOESM1_ESM.docx]

**Supplementary file to: Treating SOD1-ALS with tofersen results in non-progressive chronic ALS**

**The method on the collection of quantitative muscle strength testing.**

Quantitative muscle strength was assessed using a Jamar Plus+ Digital Hand Dynamometer, a Jamar Digital Pinch Gauge and a Lafayette Hand-held Dynamometer. For upper limb measurements, participants were seated on a bench with their feet resting on the floor and the elbow positioned at 90° flexion. For the lower limb measurements, the participants remained seated with their legs unsupported and the knee flexed at 90° without additional support from the hands. Three consecutive measurements were obtained for each muscle group, and the mean value was used for analysis. All measurements were conducted by the same physiotherapist to ensure procedural consistency. The results are presented in kilograms (kg).

**Methods used for the 10MWT, FTSTS, TUG, and 10-step stair climb tests.**

Functional performance was evaluated using the 10-Metre Walk test (10MWT), the Five Times Sit-to-Stand Test (FTSTS), the Timed Up and Go Test (TUG), and the 10-Step Stair Climb test.
For the 10MWT, participants were already walking at their self-selected pace when crossing the starting line (0 m), and timing stopped when they crossed the 10 m line. Appropriate assistive devices were permitted and used consistently across repeated assessments. In one case, both comfortable and maximal walking speeds were recorded. Two trials were performed, and the best result was used for analysis.
For the FTSTS, participants stood up from a 47 cm high chair with armrests and were instructed to complete five repetitions of sit to stand as quickly as possible at their maximal speed. In general, participants were instructed not to use their hands for support when standing up. However, in Case 3, the use of armrests was permitted, as the test could not otherwise be possible, and this approach was maintained consistently in subsequent assessments. Two trials were performed, and the best time was used for analysis.
The TUG test was performed according to standardized guidelines. Participants were instructed to walk at a comfortable speed unless otherwise indicated. Specifically, Case 3 completed the test at a comfortable walking speed, whereas Case 4 was instructed to walk at a maximal speed, as he was deemed capable of doing so safely. Three trials were conducted for each participant, and the best result was used for analysis.
For the 10-step stair climb test, participants ascended and descended through a 10-step flight without using the handrail. The test was performed at both comfortable and maximal speeds.
